# Success rate of artificial insemination, reproductive performance and economic impact of failure of first service insemination: a retrospective study

**DOI:** 10.1186/s12917-022-03325-1

**Published:** 2022-06-14

**Authors:** Belege Tadesse, Abadi Amare Reda, Nuredin Teshale Kassaw, Wedajo Tadeg

**Affiliations:** 1 School of Veterinary Medicine, Wollo University, Dessie, Ethiopia; 2 Kombolcha College of Agriculture Affiliated to Wollo University, Kombolcha, Ethiopia

**Keywords:** AI, Cows, Dairy cattle, Economic loss, Herd management, Reproductive performance

## Abstract

**Background:**

A retrospective cohort study using a 10 year artificial insemination (AI) and cow reproductive performance data was conducted to study the success rate of AI; associations between effectiveness of AI and breed, AI season and, number of service per conception, and economic impact of failure of FSC in Dessie town, Dessie zuria and Kutaber districts. A total of 3480 dairy cows’ AI and reproductive performance records which were performed between 2003 and 2013 in the three selected districts of South Wollo were used. The economic losses and costs for cows that failed to conceive at their first AI associated with the larger number of days open were estimated.

**Result:**

The prevalence of conception has a statistically significant difference between breeds of cows (*P* = 0.019). The non-return rate for first service was 58.54%. The median days to first service (DFS), inter-service interval (ISI) and gestation length (GL) were 126, 30 and 278 days respectively. Whereas, the mean + SD days open, calving interval (CI), number of inseminations (NOI) and number of services per conception (NSPC) were 147.2 ± 60.26, 424.5 ± 60.55, 1.14 ± 0.38 and 1.15 ± 0.39 respectively. Based on AI season there was a significant difference in conception between winter and spring (*P* = 0.021). There is a 45.04 days extension in the mean calving to conception interval in cows that did not conceive at their first AI but conceived by 2nd and 3rd AI than in cows that did conceive at their first AI. A total of 21,665.3 ETB extra costs was spent on reproductive treatment and other management for cows that failed to conceive at their first AI but conceived by second and third service. In cows that did not conceive totally the owner losses on average 473.7 ETB per cow per day extra costs until the cows will be culled.

**Conclusion:**

Therefore to increase the conception rate and decrease the economic loss the owners of the dairy cows should supervise the cows regularly and should be trained on how to identify cows on estrous, the AI technicians should be trained to conduct the AI service accurately.

**Supplementary Information:**

The online version contains supplementary material available at 10.1186/s12917-022-03325-1.

## Background

Ethiopia has the largest livestock population in Africa, even if its productivity remains low [1]. From the total cattle population, 99.4, 0.5 and 0.1% were indigenous, cross and exotic breeds [2] respectively. In order to improve the low productivity of local cattle, cross breeding of these indigenous breed with highly productive exotic cattle have been considered a realistic solution [3]. Nowadays, AI is recognized as the best technique for increasing reproductive capacity and has received widespread application in farm animals in Ethiopia [4].

Conception at the first service (FSC) after calving is crucial to improve the reproductive performance in dairy cows to increase the profit [5]. The success of FSC has been reported in a range between 26.7 and 50.7% in previous studies [6, 7]. A decrease in the first service conception results in an increase in the numbers of insemination, number of days open, feeding cost, culling loss, and replacement heifers cost [8, 9]. Therefore, identification of factors that potentially limit the success of FSC is useful to improve reproductive performance in dairy cows.

Several factors like parity, AI season, calving to first service interval, and peripartum disorders (dystocia, metritis, and retained placenta) have been decreasing the efficiency of FSC [6, 7, 10]. According to Quintela et al. [11] higher milk yield (>39 kg/d), genetic values and cow parities (four or greater) were associated with a higher risk of a low FSC rate in the west-central region of France [11]. Clinical ketosis, metritis, retained placenta, stillbirth, dystocia and birth of twins were associated with a moderate decrease in FSC rate [5, 12, 13]. According to Rearte et al. [14] report in northwest Spain, the higher risk of a low FSC rate is associated with short calving to first AI intervals, dystocia, parity of five and postpartum disorders autumn calving.

According to Müller-Sepúlveda et al. [15], the province, number of cows in the herd, experience of the inseminators and type of insemination affects the success of the pregnancy. The success of AI is also influenced by endometrial thickness, artifcial insemination timing, insemination frequency, and ovarian stimulation protocols [16]. According to Bastin et al. [17], the success of AI was affected by the body condition of cows.

In Ethiopia dairy farmers plan to produce one calf per cow per year to maximize milk production and guarantee dairy herd replacement. However, most of the farms lack improved breeding programmes, nutritional strategies and data management strategies. In a dairy production good herd management practices are very important to know the reproductive performance of cows and also assist decision-making process and economic evaluation [18, 19]. Low pregnancy rates results in a reduction in milk production and calves born per year, which reduces the economic profitability of the dairy farms and the country [20].

AI has a main importance in improving local breeds in our country to increase the milk production and the total gain from dairy cows. The efficiency of AI were reported by different researchers ranging from 48.1 to 86.4% in different parts of Ethiopia [21–26] However, the efficiency of first service insemination and impact of the AI has not been well-documented in Ethiopia. Thus identification of risk factors limiting FSC in dairy herds, determining the reproductive efficiency and success rate of AI and estimating the economic impact of the failure of FSC might provide useful information for dairy farmers. Therefore, this study was conducted with the objective of assessing association of FSC with some risk factors, determining the reproductive efficiency and success rate of AI, estimating the non-return rate for each service and estimating the economic impacts of failure of FSC.

## Methods

### Description of the study area

**Fig. 1:**
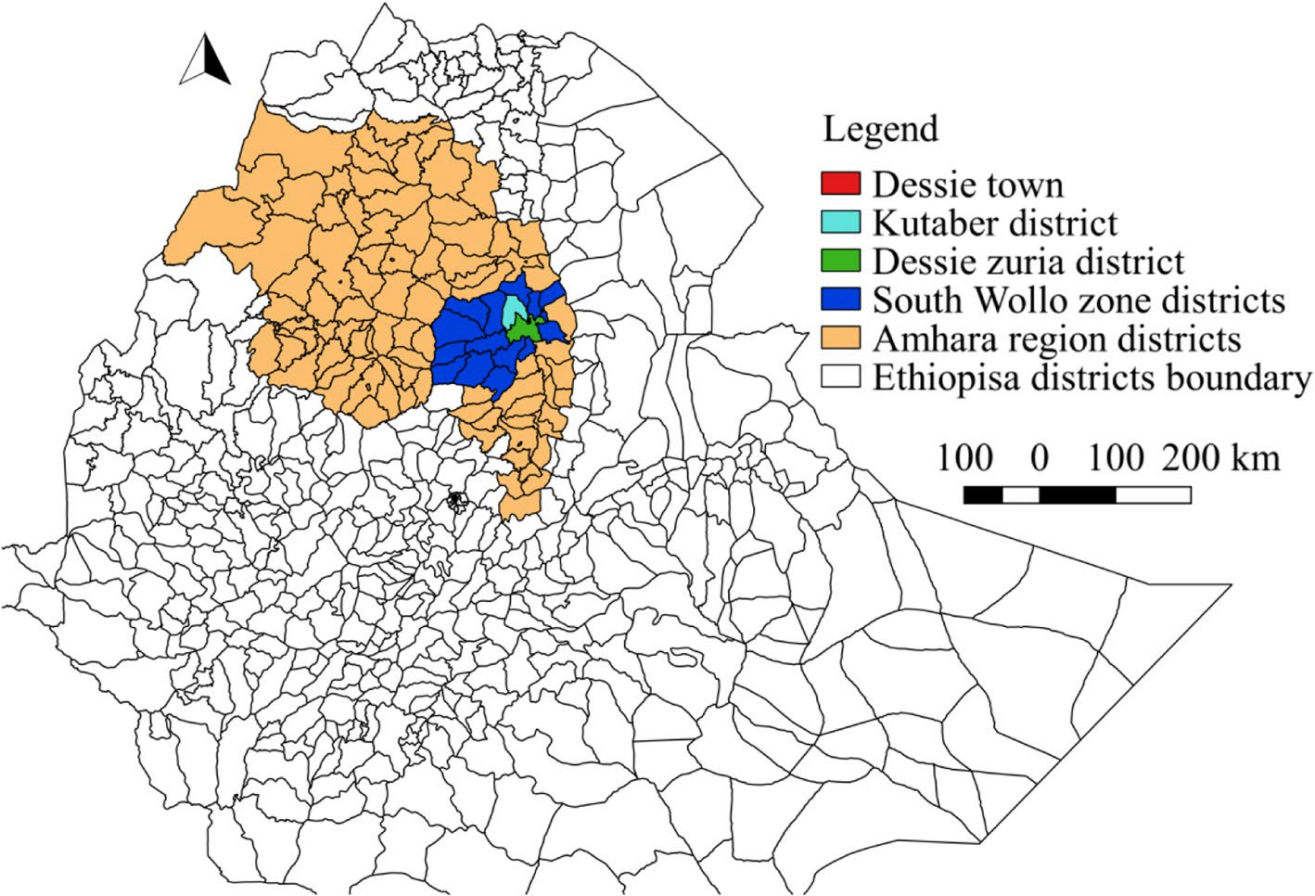
Map of the study areas (Mapped by using QGIS 2.18.28)

A 10 year AI and pregnancy diagnosis routine record book of Dessie town, Dessie zuria district and Kutaber districts (Fig. 1) were obtained from South Wollo Zonal Liquid Nitrogen Production and Semen Distribution Centre (SWLNPSDC), Dessie, Ethiopia. Dessie is located in the north eastern part of the country at a distance of 401 km north of Addis Ababa. It is placed at latitude and longitude of 11′8°N and 39′38°E respectively with an altitude range of 2470 to 2550 m above sea level. The area has an average annual rainfall of 1145 mm and a mean annual temperature of 15.2 °C. Both crop and livestock production is the main farming system of the districts. The total cattle population of the study districts was 194,889 [27].

### Study design

A retrospective cohort study design using a 10 year AI and cow reproductive performance data was conducted to study the success rate of AI, associations between effectiveness of AI and breed, AI season, number of service per conception, and economic impact of failure of FSC.

### Description of the data

A 10 year retrospective AI after heat detection and reproductive performance data of the three districts was obtained from SWLNPSDC. All the AI and reproductive performance data recorded in the three districts within the 10 years (from 2003- 2013) was used in this study. During obtaining the data consent was taken with the SWLNPSDC to use the data for this scientific study. The data was comprised of 3480 dairy cows’ AI and reproductive performance records which were performed between 2003 and 2013 in the selected districts of South Wollo zone. The data includes the following reproductive performance variables: owners name and address, cow ID, cow breed (local = 514, cross = 2966), Sire ID (*n* = 26), last calving date, first AI date (*n* = 3034), second AI date (*n* = 406) and third AI date (*n* = 40) and their corresponding insemination bull number; pregnancy diagnosis date and outcome of AI, calving date and calf sex.

### Data exploration and editing

Data exploration and editing was done using Microsoft Excel. Several new variables as measures of reproduction efficiency and performance were derived from the aforementioned original data. These includes number of services per conception (NSPC), postpartum conception (PC), calving interval (CI), gestation length (GL), Inter service interval (ISI), days to first service (DFS), number of inseminations (NOI), calving type, fertility level, parity, season of AI and season of calving.

### Measure of conception rate and reproductive efficiency

The conception rate was estimated by dividing conceived cows by the number of inseminated cows during that period. Different factors that may affect the conception rate were also assessed. The days open, calving interval, services per conception, days to first service and inter-calving intervals were also estimated from the data.

### Evaluation of the economic impact of failure of first service conception

The costs associated with the success or failure of first service conception by AI includes the costs of AI and pregnancy diagnosis (PD) both for the cows that conceived and those that failed to conceive at their first AI, and the costs of extra management procedures for cows that failed to conceive at their first AI, incurred because of a higher number of days open than for cows that did conceive at their first AI [28]. The cost of AI and PD was calculated using the total costs of semen, AI technician, and PD until conception occurred. The extra economic losses and costs for cows that failed to conceive at their first AI comprised the costs of replacement heifers, value of extra feed fed in additional days, value of extra labor used for management of animal, value of extra breeding, value of calf loss and value of milk loss associated with the larger number of days open.

The milk loss due to longer number of days open was estimated based on the average milk yield of that cow, number of days from first fail of AI to conception and the price of milk per litter in the town. The Feed cost per cow was estimated based on the recorded daily consumption and local market price of the feed. A calf price was set based on the value of a calf in the local market. The costs and losses from different factors were estimated using the following formulas according to Ill Hwa Kim and Jae Kwan Jeon [29].

1. Mean number of Extra days of calving to conception = (Total number of days from calving to conception in cows conceived by 2nd and 3rd service AI – total number of days from calving to first service AI)/ the number of cows conceived by 2nd and 3rd service AI.
2. Replacement cost = Replacement cost per cow/day*extra days of calving to conception = [(difference b/n the price of replaced cow and culled cow*% of culling due to infertility)* extra days of calving to conception /calving interval].
3. Calf price = Calf price per cow/day* extra days of calving to conception = (price of calf/ calving interval)* extra days of calving to conception
4. Cost of nutrition = Cost of nutrition per cow/day*extra days of calving to conception
5. Labor cost = extra days of calving to conception*daily labor cost
6. Milk cost = extra days of calving to conception*average daily milk yield of that cow*price of milk/litter
7. AI cost = number of insemination*cost of single insemination
8. Palpation (PD) cost = no. of PD*single PD cost

### Data management and analysis

The collected data were entered into Microsoft Excel spread Sheet, edited and analyzed using Stata Version 13. Accordingly, descriptive statistics such as percentages and frequency distribution were used to determine the efficiency of pregnancy with different factors and the association of conception with different factors has been tested using multiple logistic regressions. A value of *p* < 0.05 was considered as significant. The economic losses were analyzed descriptively.

## Results

### Description of study cows profile

For studying the prevalence of pregnancy, a total of 3480 artificially inseminated dairy cows (514 local breed and 2966 cross breed cows) from 2003 to 2013 were retrospectively collected and used. From the total of 2052 conceived cows 1776 (86.55%) were inseminated only once, whereas 276(13.45%) were inseminated more than once (Table 1). Among the 2052 conceived cows 81 (3.92%) encountered abortion.

**Table 1:** Conception rate with different factors

Variables		Frequency	Conceived/pregnant N(%)	Chi-square	*P* value
**Season of AI**	Winter	1008	606 (60.12)	2.538	0.468
	Spring	877	498 (56.14)		
	Summer	867	512 (59.10)		
	Autumn	728	436 (59.89)		
**Breed of cows**	Local	514	279 (54.28)	0.20	0.019
	Cross	2966	1773 (59.757)		
	Overall	3480	2052 (58.97)		
**No. of services**	1	3480	1776 (51.03)	2.99	0.224
	2	406	248 (61.10)		
	3	40	28 (70.00)		
**Overall**		3480	2052 (58.97)		

There was no significant variation in conception between seasons of AI and number of services per conception (*P* > 0.05); but there was a statistically significant difference between breeds of cows (*P* = 0.019) in which a higher prevalence was achieved in cross breed cows. Based on the number of services per conception, those cows inseminated for the third time have high conception rate (70%) (Table 1).

### Non - return rate

The non return rate for each service and the AI submission rates in <85 days postpartum were indicated in Table 2. The non-return rate for first service was 58.54%.

**Table 2:** Non return rate to each service

Number of services	Total inseminated	Number conceived (NRR)
**1**	3034	1776 (58.54%)
**2**	406	248 (61.10%)
**3**	40	28 (70.00%)
**AI submission rates **<**85 days postpartum**	995	149 (14.9%)

### Reproductive parameters

The median DFS, ISI and GL were 126, 30 and 278 days respectively. Whereas, the mean + SD days open, CI, NOI and NSPC were 147.2 ± 60.26, 424.5 ± 60.55, 1.14 ± 0.38 and 1.15 ± 0.39 respectively (Table 3).

**Table 3:** Summary statistics of continuous and count reproductive variables/parameters

Variables	No of cows	Minimum	1st quartile	Median	Mean ± SD	3rd quartile	Maximum
**DFS (days)**	995	35.0	97.0	126.0	140.3 ± 60.17	168.0	598.0
**ISI (days)**	446	4.00	21.00	30.00	39.73 ± 23.72	56.75	150.00
**GL (days)**	1883	253.0	273.0	278.0	277.5 ± 6.34	282.0	295.0
**Days open**	627	46.0	101.0	134.0	147.2 ± 60.26	179.0	416.0
**CI (days)**	571	329.0	379.0	412.0	424.5 ± 60.55	455.0	699.0
**NoI**	3480	1.00	1.00	1.00	1.14 ± 0.38	1.00	3.00
**NSPC**	1883	1.00	1.00	1.00	1.15 ± 0.39	1.00	3.00

*DFS* Days from calving to first service, *ISI* Inter service interval, *GL* Gestation length, *CI* Calving interval, *NoI* Number of insemination, *NSPC* Number of service per conception

**Fig. 2:**
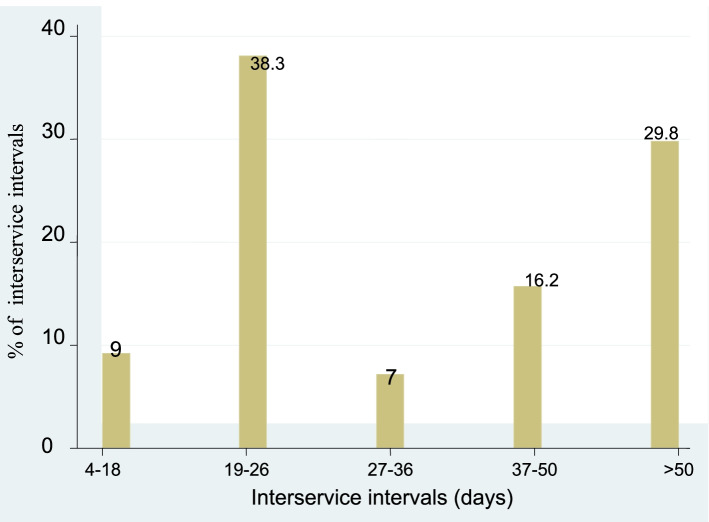
Interservice intervals

From the calculated inter service intervals 9 and 38.3% were distributed in the range of 4 to 18 days and 19 to 26 days respectively. Whereas 30% of the ISI falls in greater than 50 days and 61.7% had greater than 26 days ISI which indicates that there was a gap in the ability of estrous detection (Fig. 2).

The estimated mean gestation length varies significantly (*p* < 0.005) between calving type, NSPC, AI season and calving season. Whereas, estimated mean calving interval varies significantly (*p* < 0.001) between breed/genotype, fertility and NSPC. The estimated mean postpartum day varies significantly (*p* < 0.005) between breed, fertility and NSPC (Table 4).

**Table 4:** Association of gestation length, calving interval and postpartum days with other variables

		Gestation length in days (*N* = 2052)		Calving interval in days (*N* = 2052)		Postpartum days (*N* = 2052)	
Variables	Categories	EMM (days) ± SE	*p*-value	EMM (days) ± SE	*p*-value	EMM (days) ± SE	*p*-value
**Genotype**	Local	278.1 ± 0.3962	0.1073	439 ± 6.79	0.0259	162 ± 6.36	0.0118
	Crossbred	277.4 ± 0.1572		422 ± 2.72		145 ± 2.59	
**Calving type**	PTC	272.8 ± 0.1206	<0.0001	421 ± 3.43	0.1255	148 ± 3.42	0.7518
	FTC	282.9 ± 0.1311		429 ± 3.75		147 ± 3.74	
**Fertility**	Normal	277.5 ± 0.4516	0.8138	369 ± 3.25	<.0001	90.9 ± 3.02	< 0.0001
	Subfertility	277.6 ± 0.3240		453 ± 2.33		176.8 ± 2.19	
**Parity**	Prim/Heifer	277.0 ± 0.3759	0.2287	–	–	–	–
	Multiparous	277.6 ± 0.2599		425 ± 2.53		147 ± 2.41	
**NSPC**	First AI	277.1 ± 0.1558	< 0.001	419 ± 2.74	< 0.001	142 ± 2.72	< 0.001
	Second AI	279.8 ± 0.4153		450 ± 6.26		173 ± 6.20	
	Third AI	278.1 ± 1.2094		461 ± 17.14		198 ± 16.98	
**AI Season**	Summer	278.1 ± 0.2679	< 0.001	420 ± 4.82	0.3924	143 ± 4.51	0.6715
	Spring	278.5 ± 0.2904		428 ± 4.93		149 ± 4.73	
	Winter	276.8 ± 0.2917		430 ± 5.12		150 ± 4.83	
	Autumn	276.2 ± 0.3138		420 ± 5.48		148 ± 5.31	
**Calving season**	Summer	278.8 ± 0.2867	< 0.001	428 ± 4.83	0.4577	150 ± 4.81	0.3757
	Spring	276.7 ± 0.2946		429 ± 5.12		153 ± 5.09	
	Winter	275.9 ± 0.3086		422 ± 5.55		146 ± 5.52	
	Autumn	278.1 ± 0.2669		419 ± 4.87		141 ± 4.84	

*EMM* estimated marginal means

Days to first service varies significantly between breed and fertility, whereas inter service interval varies significantly between fertility, NSPC, calving season and AI season. The estimated means of NSPC varies significantly between calving type, fertility and parity (*p* < 0.001) (Table 5).

**Table 5:** Association days to first service, inter-service interval and number of service per-conception with other variables

		Days to First Service (*N* = 2052)		Inter-service interval (*N* = 276)		NSPC (*N* = 2052)	
Variables	Categories	EMM (days) ± SE	*p*-value	EMM (days) ± SE	*p*-value	EMM (days) ± SE	*p*-value
**Genotype**	Local	155 ± 4.86	0.0015	37.4 ± 3.04	0.4025	1.14 ± 0.025	0.555
	Crossbred	138 ± 2.06		40.1 ± 1.21		1.15 ± 0.0098	
**Calving type**	PTC	142 ± 3.29	0.3020	43.5 ± 2.54	0.1331	1.11 ± 0.0123	<.0001
	FTC	137 ± 3.60		38.6 ± 2.06		1.19 ± 0.0134	
**Fertility**	Normal	89.2 ± 2.56	< 0.0001	25.4 ± 5.75	0.0006	1.07 ± 0.0316	<.0001
	Subfertility	167.3 ± 1.85		46.7 ± 1.98		1.27 ± 0.0227	
**Parity**	Prim/Heifer	–	–	37.2 ± 3.23	0.059	1.14 ± 0.026	0.0427
	Multiparous	140 ± 1.91		44.4 ± 1.99		1.20 ± 0.018	
**NSPC**	First AI	142 ± 2.67	0.1469	–	<.0001	–	–
	Second AI	133 ± 6.11		36.0 ± 1.46		–	–
	Third AI	114 ± 16.72		79.1 ± 4.24		–	–
**AI Season**	Summer	137 ± 3.49	0.7656	35.2 ± 1.99	0.001	1.15 ± 0.0169	0.8676
	Spring	141 ± 3.72		34.7 ± 2.18		1.14 ± 0.0183	
	Winter	142 ± 3.81		45.8 ± 2.18		1.16 ± 0.0184	
	Autumn	142 ± 4.42		45.6 ± 2.53		1.14 ± 0.0198	
**Calving Season**	Summer	142 ± 4.63	0.5992	41.5 ± 3.09	0.002	1.15 ± 0.0182	0.8784
	Spring	144 ± 4.91		45.9 ± 3.27		1.15 ± 0.0187	
	Winter	137 ± 5.32		47.2 ± 3.62		1.14 ± 0.0195	
	Autumn	136 ± 4.66		32.2 ± 2.75		1.16 ± 0.0169	

*EMM* estimated marginal means

### Association of conception with season of AI, breed of cows and parity

Based on multiple logistic regression analysis conception of cows was statistically significantly different between breed of cows (*p* = 0.030); whereas, there was no any significant difference in conception based on season of AI and parity (*p* > 0.05) (Table 6). Cross breed cows have a higher probability of conception than local breed.

**Table 6:** Multiple logistic regression result indicating association of some risk factors with conception

Variable		OR (95%CI)	*P* value
**Cow Breed**	Local	Ref.	0.019
	Cross	1.25 (1.04-1.51)	
**AI season**	Winter	Ref.	–
	Spring	0.58 (0.36-0.92)	0.021
	Summer	0.97 (0.65-1.47)	0.89
	Autumn	0.86 (0.55-1.35)	0.51
**Parity**	Primiparous	Ref.	0.342
	Multiparous	1.11 (0.89-1.39)	

### Economic impact of failure of first service conception

The culling rate owing to infertility in cows that did not conceive at their first AI was 80.2% (279/348), whereas no cows were culled because of infertility if they did conceive at their first AI (0/1776).

The analysis showed that 41.03% of the cows were censored because they were sold, died, or had not conceived until the end of the study years. There is a 45.04 days extension in the mean calving to conception interval in cows that did not conceive at their first AI but conceived by 2nd and 3rd AI than in cows that did conceive at their first AI.

The expense of reproductive treatment required until conception in cows that did or did not conceive at their first AI was shown on Table 7. Cows that failed to conceive at first AI (i.e. conceived by second and third service) required an extra 137.5 ETB due to extra semen and palpation cost than cows that did conceive at their first AI. A total of an additional expense of 21,402.8 ETB was incurred for other reproductive management procedures required to achieve conception (replacement heifers, nutrition, calf price, milk, and labor) in cows that failed to conceive at their first AI (Table 8). Thus, a total of 21,665.3 ETB extra costs was spent on reproductive treatment and other management for cows that failed to conceive at their first AI but conceived by second and third service. In cows that did not conceive totally the owner losses on average 473.7 ETB per cow per day extra costs until conception (Table 8).

**Table 7:** Costs of AI and PD per cow required to achieve conception in cows that did or did not conceive at their first AI (ETB)

Item	Unit	Value (ETB) /dose	Cows that did not conceive at first AI but conceived by 2nd &3rd AI (***n*** = 276)	Cows that did conceive at first AI (***n*** = 1776)
AI (semen, technician, straw)	1 straw	75	2.1straw*75 = 157.5	1 straw*75 = 75
PD	Number	17/50	2.1 palpation*50 = 105	1 palpation*50 = 50
Total			262.5	125

*ETB* Ethiopian birr, 1USD = 40ETB (Ethiopian birr) during the study period

**Table 8:** Additional expenses for management procedures in cows that failed to conceive at their first AI, incurred due to a larger number of days open

Item	Additional costs per cow/day in cows that did not conceive by first AI	Additional costs in cows conceived by second and third AI
**Replacement**	Difference between the value of cull cows (30000) and replacement heifers (cows) (50000)* Cost of replacement per cow/day =(20000*80.2%^a^/424.55^b^) = 37.8ETB	Mean extra days of calving to conception * Cost of replacement per cow/day = 45.04 days*37.8ETB = 1702.5 ETB
**Nutrition**	` Cost of nutrition per cow/d: 140ETB	Extra days of calving to conception * Cost of nutrition per cow/d =45.05 days*140 ETB = 6370ETB
**Calf price**	Calf price per cow/d: (2500ETB/424.55 days^b^): 5.9ETB	Extra days of calving to conception * Calf price per cow/d =45.05 days*5.9ETB = 265.8ETB
**Labor**	Labor cost per cow/d: 50ETB	Extra days of calving to conception * Labor cost per cow/d = 45.05 days * 50ETB = 2252.5ETB
**Milk loss**	Milk lost per cow/d: (12Litter*20ETB) = 240ETB	Extra days of calving to conception * Milk lost per cow/d **=** 45.05 days*240ETB = 10812ETB
**Total**	473.7ETB	21,402.8ETB

^a)^ Culling due to infertility in cows that failed to conceive at first service: 279/348 (80.2%)

^b)^ Calving interval in this study

## Discussion

In the current study breed wise the conception rate were 54.28 (279/514) and 59.757 (1773/2966) in local and cross breed cows respectively. This finding is lower as compared to the overall conception rate of 74.67 and 64.8% in dairy cows in and around Kombolcha town [22] and in Dairy Cows in and Around Bishoftu [23] in Ethiopia. This result is also lower than the report of Shiferaw *et al*. [24], Jemal *et al*. [25], Arthur *et al*. [26], Balachandran [30], Basuro *et al.* [31], and who reported a pregnancy rate of 65.6, 62.1, 84.66, 86.4 and 63%-71% respectively. Whereas it is higher than the 48.1% conception rate reported by Engidawork [21] in selected districts of Harari region. The difference in the conception rate could be due to difference in the composition of cows, number of cows, production system, type of semen, environment, inseminator potential and other management conditions.

Cross breed cows had 1.25 (CI = 1.04-1.51) times higher odds of occurrence of conception than local breed cows. This agrees with the finding of Befkadu et al. [22] and Yehalaw et al. [23], who reported a higher conception rate in cross breed cows in dairy cows in and around Kombolcha town in Ethiopia. The abortion rate found in the current study is 3.92%, which is higher than the 1.4% reported by Lobago et al. [32] in Sellale, Central Ethiopia.

The non -return rate at first insemination in the current study was 86.55%. The result obtained in this study is higher than the 48.1% [21], 75% [33] and 84.03% [34] reported in Hareri, North Gondar, showa and North Gondar zone respectively. The variability on the value of non return rate might be due to difference in semen handling practices, AI technicians, breed, geography and differences in semen quality used for insemination.

The mean number of service per conception in this study was 1.15 ± 0.39. This is lower than the 1.6 services per conception reported in central highlands of Ethiopia [35] and Harari [21]. It is also lower than the 1.88 [8], 1.7 [21] and 2.2 [22] reported in north Gonder zone, in and around Zeway and Eastern Lowlands of Ethiopia respectively. The number of service per conception higher than 2.0 were considered as poor [36]. Thus, the result found in the current study can be considered as good.

The estimated mean NSPC varies significantly between calving type, fertility and parity. The finding was in agreement with findings reporting the significant effect of parity of dam on number of service per conception [37–39]. However, according to the study reported by Engidawork et al. [33], Number of services per conception was not significantly affected by previous calving season and parity. NSPC was dependent on a large number of factors such as the oestrus display, oestrus detection, timing of service, sire fertility and sperm quality, subclinical diseases, and management features. Other studies are needed to investigate all aspects of increased NSPC.

In the current study 9 and 38.3% of the ISI were distributed in the range of 4 to 18 days and 19 to 26 days respectively. In addition 29.5% of interservice intervals were greater than 50 days. This is higher than the report of Softic et al. [40], who reported that a total of 9.6% of interservice intervals were longer than 48 days. Remnant et al. [41] reported that ISI of 19–26 days indicated that this period is the true latent distribution for the ISI with the optimal reproductive outcome, suggesting day-22 with the increased probability of conception [41]. However in our study 75% of the cows had 56.73 ISI and the mean is 39.73 ± 23.72 days which indicates the need of targeted monitoring of cows in order not to miss cows on estrous. This shows that there was a problem in the detection of cows on oestrous.

The mean (± SD) CI of 424.5 ± 60.55 in the current study is higher than the report of 385 day by Softic et al. [40] and 12.6 months [42] in Dairy Farms in Una-Sana Canton, Bosnia and Herzegovinathe and Norwegian Red cattle respectively. However the CI is calculated retrospectively and represents the sum of all previous reproductive measures, it could be influenced by wide individual variations within the cows included in the study. Since there was a difference in the management, feed, and blood levels of cows.

The median DFS in this study was 126 days with variations between individual cows. This is highly greater than the 62.5 days reported by Softic et al. [40] in Dairy Farms in Una-Sana Canton, Bosnia and Herzegovinathe. It is also lower as compared to the report for Norwegian Red cattle (85.3 days, SD ± 41.9) [43]. The variations in DFS between individual cows and different studies can be explained by several factors such as nutrition [35, 42, 44], endometritis [44], and poor oestrus detection. According to Elkjær et al. [33, 45] report uterine infection was associated with poor reproductive performance.

The median and mean days open in this study were 134 and 147.2 ± 60.26 respectively. The median days open in this study was higher than the 101 days open reported by Softic et al. [40] in Una-Sana Canton. The high median and mean days open in this study could be due to ability of detection of estrous, quality of semen and management of semen and cows. To reduce the mean open days, strengthening the heat detection ability and timed AI could be an alternative cost-effective measure. Cows with chronic reproductive problems could also be culled from the dairy herd and replaced by other cows [46, 47].

The economic loss/extra cost due to the failure of FSC in the current study was 21,665.3 ETB due to extra costs of reproductive treatment and other management for cows that failed to conceive at their first AI but conceived by second and third service. It is found that a greater economic loss was resulted from management of cows (replacement heifers, nutrition, calf price, and labor) necessitated by the larger number of days open (81 days) than reproductive treatment (including semen, and palpation). In a previous study reported by Ill Hwa Kim and Jae Kwan Jeon [29] a total economic loss of $622.40 per animal was reported due to the failure of FSC in Korea. In another study in cows that needs three or more inseminations per conception the profit was decreased by >$205/year per cow [9].

The findings of the current and the previous studies showed that larger numbers of services per conception results in greater economic loss. The magnitude of the economic loss may differ depending on the reproductive efficiency and the amount of other expenses associated with management on dairy cows with extra days open [48]. The estimate of economic loss due to the failure of FSC in the current study and in the previous reports showed that dairy managers and owners should consider the impact of failure of FSC and the requirement to adopt strategies to improve FSC in dairy herds.

## Conclusions

Relatively a moderate conception rate was encountered in this study. The conception rate differs between breed of cows and season of AI. Relatively higher average days to service and non-return rate to first conception were estimated. A total of 21,665.3 ETB was incurred on cows that failed to conceive at their first AI but conceived by second and third service. Whereas in cows that did not conceive totally the owner losses on average 473.7 ETB per cow per day extra costs until the cow will, return to estrous or will be culled. Therefore to increase the conception rate and the economic loss the owners of the dairy cows should supervise the cows regularly, the owners should be trained on how to identify cows on estrous, the AI technicians should be trained to conduct the AI service accurately, the government should actively involved in the improvement of the local breeds and a cost-benefit analysis should be implemented in dairy farm activity.

## Supplementary Information

**Additional file 1.** Definition of Terms.

## Data Availability

The datasets generated and/or analyzed during the current study are not publicly available because the data is huge and we use it for further works, but are available from the corresponding author on reasonable request.

## Abbreviations

AI: Artificial insemination
CI: Calving interval
DFS: Days to First Service
ETB: Ethiopian birr
GL: Gestation Length
ISI: Inter Service Interval
PD: Pregnancy Diagnosis
NOI: Number of inseminations
NSPC: Number of services per conception
PC: Postpartum Conception
SWLNPSDC: South Wollo Zonal Liquid Nitrogen Production and Semen Distribution Centre

## References

[1] Yohaness S, Tenhagn BA, Bekana M, Teshager K (2003). Reproductive performance of crossbred dairy cows in different production systems in the central highlands of Ethiopia. Trop Animal Hlth Prod.

[2] EASE, Ethiopian Agricultural Sample Enumeration (2003). Statistical report on Farm Management Practice, livestock and farm implements part II.

[3] Tadesse B (2002). Calf sex ratios in artificial insemination and natural mated female cross breed daily herd. Proceedings of the 13th Annual Conference of the Ethiopia Society of Animals Production Addis Ababa Ethiopia.

[4] Gizaw Y, Bekana M, Abaynesh T. Major reproductive health problems in small holder daily production in and around Nazareth town, Central Ethiopia. J Vet Med Animal Hlth. 2007;5(4):112–5.

[5] Inchaisri C, Hogeveen H, Vos PL, van der Weijden GC, Jorritsma R (2010). Effect of milk yield characteristics, breed, and parity on success of the first insemination in Dutch dairy cows. J Dairy Sci.

[6] Siddiqui MAR, Das ZC, Bhattacharjee J (2013). Factors affecting the first conception rate of cows in smallholder dairy farms in Bangladesh. Reprod Domest Anim.

[7] Tillard E, Humblot P, Faye B (2008). Postcalving factors affecting conception risk in Holstein dairy cows in tropical and subtropical conditions. Theriogenology..

[8] Chang YM, Andersen-Ranberg IM, Heringstad B, Gianola D, Klemetsdal G (2006). Bivariate analysis of number of services to conception and days open in Norwegian Red using a censored threshold-linear model. J Dairy Sci.

[9] González-Recio O, Pérez-Cabal MA, Alenda R (2004). Economic value of female fertility and its relationship with proft in Spanish dairy cattle. J Dairy Sci.

[10] Grimard B, Freret S, Chevallier A (2006). Genetic and environmental factors influencing first service conception rate and late embryonic/foetal mortality in low fertility dairy herds. Anim Reprod Sci.

[11] Quintela LA, Peña AI, Taboada MJ (2004). Risk factors for low pregnancy rate in dairy cattle: A retrospective study in the north west of Spain. Arch Zootec.

[12] Fourichon C, Seegers H, Malher X. Effect of disease on reproduction in the dairy cow: a meta-analysis. Theriogenology. 2000;53:1729–59. 10.1016/s0093-691x(00)00311-3 10968418

[13] Ferguson JD, Skidmore A. Reproductive performance in a select sample of dairy herds. J Dairy Sci. 2013;96(2):1269–1289. 10.3168/jds.2012-5805 23182352

[14] Rearte R, LeBlanc SJ, Corva SG, de la Sota RL, Lacau-Mengido IM, Giuliodori MJ (2018). Effect of milk production on reproductive performance in dairy herds. J Dairy Sci.

[15] Müller-Sepúlveda A, Foerster C, Arriagada G, Juan-Eduardo Silva JE, Ortiz M (2020). Factors that affect the success of artificial insemination in cattle of small farmers in the O’Higgins region of central Chile. RovFCA UNCuyo.

[16] Wang X, ZhanY SHL, Wang LT, Li XF, Wang F, Wang YL, Li QC (2021). Factors affecting artificial insemination pregnancy outcome. Int J Gen Med.

[17] Bastin C, Loker S, Gengler N, Sewalem A, Miglior F (2010). Genetic relationships between body condition score and reproduction traits in Canadian Holstein and Ayrshire first-parity cows. J Dairy Sci.

[18] Eaglen SAE, Coffey MP, Woolliams JA, Wall E (2013). Direct and maternal genetic relationships between calving ease, gestation length, milk production, fertility, type, and lifespan of Holstein-Friesian primiparous cows. J Dairy Sci.

[19] Miglior F, Fleming A, Malchiodi F, Brito LF, Martin P, Baes CF (2017). A 100-year review: identification and genetic selection of economically important traits in dairy cattle. J Dairy Sci.

[20] Softic A, Asmare K, Granquist EG, Godfroid J, Fejzic N, Skjerve E (2018). Serostatus of Brucella spp., Chlamydia abortus, Coxiella burnetii and Neospora caninum in cattle in three cantons in Bosnia and Herzegovina. BMC Vet Res.

[21] Engidawork B (2018). Artificial Insemination Service Efficiency and Constraints of Artificial Insemination Service in Selected Districts of Harari National Regional State, Ethiopia. Open J Animal Sci.

[22] Befkadu Y, Tadesse B, Hamid M (2019). Efciency of Artifcial Insemination in Dairy Cows in and around Kombolcha Town, South Wollo, Ethiopia. Dairy Vet Sci J.

[23] Yehalaw B, Jemberu A, Asnake A, Wube A, Hirpa A (2018). Factors Affecting the Efficiency of Artificial Insemination in Dairy Cows in and Around Bishoftu (Debre Zeite), Oromia Regional State, Ethiopia. J Reprod Infer.

[24] Shiferaw T, Shibiru T, Cherinet M (2002). Experience on field AI management in Ethiopia. Ethio Soci Animal Prod.

[25] Jemal H, Lemma T, Bekana M (2016). Assessment of the reproductive performance of dairy cows in smallholder dairy farms using artificial insemination. Livest Res Rural Dev.

[26] Arthur GH, Noakes DE, Pearson H. Veterinary reproduction and obstetrics. Theriogenology 6th ed. Baillier Tindall UK. 1989:83–5.

[27] South Wollo Zone Agricultural Office (SWZAO). Fourth quarter report of 2019.12–14.

[28] Tenhagen BA, Drillich M, Surholt R, Heuwieser W (2004). Comparison of timed AI after synchronized ovulation to AI at estrus: reproductive and economic considerations. J Dairy Sci.

[29] Kim IH, Jeong JK (2019). Risk factors limiting first service conception rate in dairy cows and their economic impact. Asian Aust J Anim Sci.

[30] Balachandran K (1975). Artificial insemination and herd fertility level of cattle in Sri Lanka. Anim Breed.

[31] Basuro C, Martinez F, Gutierrez I (1997). Factors causing changes in the fertility of inseminated Holstein versus Zebu cows in the humid tropics. Veterinarian..

[32] Lobago F, Bekana M, Gustafsson H, Kindahl H (2006). Reproductive performances of dairy cows in smallholder production system in Selalle, Central Ethiopia. Trop Animal Hlth Prod.

[33] Engidawork B, Mekasha Y, Kebede K. Evaluation of artificial insemination service efficiency and reproductive performance of crossbred dairy cows in North Shewa zone, Ethiopia. M.Sc. Thesis, school of animal and range sciences, school of graduate studies of Haramaya University, Haramaya; 2013.

[34] Abate H. Evaluation of Artificial Insemination Service Efficiency and Reproductive Performance of F1 Friesian Crosses in North Gonder Zone, Ethiopia: M.Sc. Thesis, Alemaya University, Haramaya; 2008.

[35] Thatcher WW, Santos J, Silvestre F, Kim I, Staples C (2010). Perspective on physiological/endocrine and nutritional factors influencing fertility in post-partum dairy cows. Reprod Domest Anim.

[36] Mukasa-Mugerwa E, Tegegne A. Reproductive performance in Ethiopian Zebu (Bos indicus) Cattle: constrainst and impact on production. In 4. National livestock improvement conference, Addis Ababa (Ethiopia). IAR; 1991;1993.

[37] Enyew N, Brannang E, Rotmann OJ. Reproductive performance and herd life of crossbred dairy cattle with different levels of European inheritance in Ethiopia. In 7. Annual conference of the Ethiopian society of animal production, Addis Ababa (Ethiopia); 1999.

[38] Afewarke Y, Tegegne A, Kassa T (2001). Reproductive Performance of Crossbred Dairy Cows at Asella Livestock Research Station, Arsi, Ethiopia. Ethiopia J Animal Prod.

[39] Yifat D, Kelay B, Bekana M, Lobago F, Gustafsson H, Kindahl H (2010). Study on Reproductive Performance of Crossbred Dairy Cattle under Smallholder Conditions in and around Zeway, Ethiopia. Livest Res Rural Dev.

[40] Softic A, Martin AD, Skjerve E, Fejzic N, Goletic T, Kustura A, et al. Reproductive Performance in a Selected Sample of Dairy Farms in Una-Sana Canton, Bosnia and Herzegovina. Vet Med Inter. 2020:2190494. 10.1155/2020/2190494. 10.1155/2020/2190494 PMC7102479 32257094

[41] Remnant JG, Green MJ, Huxley JN, Hudson CD (2018). Associations between dairy cow inter-service interval and probability of conception. Theriogenology.

[42] Rodney RM, Celi P, Scott W, Breinhild K, Santos JEP, Lean IJ (2018). Effects of nutrition on the fertility of lactating dairy cattle. J Dairy Sci.

[43] Refsdal AO (2007). Reproductive performance of Norwegian cattle from 1985 to 2005: trends and seasonality. Acta Vet Scand.

[44] Gilbert RO, Shin ST, Guard CL, Erb HN, Frajblat M (2005). Prevalence of endometritis and its effects on reproductive performance of dairy cows. Theriogenology..

[45] Elkjaer K, Ancker ML, Gustafsson H (2013). Uterine bacterial flora in postpartum Danish Holstein dairy cows determined using DNA-based fingerprinting: correlation to uterine condition and calving management. Anim Reprod Sci.

[46] Macmillan K, Loree K, Mapletoft RJ, Colazo MG (2017). Short communication: optimization of a timed artificial insemination program for reproductive management of heifers in Canadian dairy herds. J Dairy Sci.

[47] Ribeiro ES, Galvao KN, Thatcher WW, Santos JEP (2012). Economic aspects of applying reproductive technologies to dairy herds. Anim Reprod.

[48] Boichard D (1990). Estimation of the economic value of conception rate in dairy cattle. Livest Prod Sci.

