# Supplementary material for: Success rate of artificial insemination, reproductive performance and economic impact of failure of first service insemination: a retrospective study
Source: BMC Vet Res. 2022 Jun 14;18:226. doi: 10.1186/s12917-022-03325-1 (PMC9199250; doi:10.1186/s12917-022-03325-1)
Supplement: Supplementary file 1 — Additional file 1. Definition of Terms. [file 12917_2022_3325_MOESM1_ESM.docx]

**Supplementary file**

**Additional file 1: Definition of Terms**

**Calving interval (CI)**: The number of days between the birth of a calf and the birth of a subsequent calf, both from the same cow.

**Inter-service interval (ISI)**: number of days between two successive services.

**Days to first service (DFS):** Number of days from last calving to first service/ becoming to heat.

**Days to calving (DC)**: is defined as the number of days from the start of joining to the day of calving.

**Days open (DO):** Number of days from calving to conception.

**Pregnancy Rate (PR):** Number of cows conceived per inseminated cows during the 10 year periods.

**Non-return rate (NRR):** This is the number of cows bred that do not come back in heat and are thus assumed to have conceived.

**Artificial insemination submission rates in < 85 days postpartum (AIS):** were defined as the percentages of cows inseminated within 85 days postpartum in cows to be bred during the same period.

**Season of calving and first AI were grouped as:**

Winter: December to February; Spring: March to May; Summer: June to August; Autumn: September to November.
